# Enriched transcription factor signatures in triple negative breast cancer indicates possible targeted therapies with existing drugs

**DOI:** 10.1016/j.mgene.2015.04.002

**Published:** 2015-05-15

**Authors:** Scooter Willis, Pradip De, Nandini Dey, Bradley Long, Brandon Young, Joseph A. Sparano, Victoria Wang, Nancy E. Davidson, Brian R. Leyland-Jones

**Affiliations:** a Avera Cancer Institute, Sioux Falls, SD, United States; b The Scripps Research Institute, Jupiter, FL, United States; c Montefiore Medical Center, Bronx, NY, United States; d Dana Farber Cancer Institute, Boston, MA, United States; e University of Pittsburgh Cancer Institute and UPMC Cancer Center, Pittsburgh, PA, United States

**Keywords:** Breast cancer, Triple negative, ER +, GSEA, NR6A1

## Abstract

**Purpose:**

Triple negative (TN) breast cancers which lack expression of the estrogen (ER), progesterone (PR), and human epidermal growth factor 2 (HER2) receptors convey a poor prognosis due in part to a lack of targeted therapies.

**Methods:**

To identify viable targets for the treatment of TN disease, we have conducted a gene set enrichment analysis (GSEA) on seven different breast cancer whole genome gene expression cohorts comparing TN vs. ER + HER2 − to identify consistently enriched genes that share a common promoter motif. The seven cohorts were profiled on three different genome expression platforms (Affymetrix, Illumina and RNAseq) consisting in total of 2088 samples with IHC metadata.

**Results:**

GSEA identified enriched gene expression patterns in TN samples that share common promoter motifs associated with SOX9, E2F1, HIF1A, HMGA1, MYC BACH2, CEBPB, and GCNF/NR6A1. Unexpectedly, NR6A1 an orphan nuclear receptor normally expressed in germ cells of gonads is highly expressed in TN and ER + HER2 − samples making it an ideal drug target.

**Conclusion:**

With the increasing number of large sample size breast cancer cohorts, an exploratory analysis of genes that are consistently enriched in TN sharing common promoter motifs allows for the identification of possible therapeutic targets with extensive validation in patient derived data sets.

## Introduction

Breast cancer is the most commonly diagnosed cancer in women (more than 230,000 women were diagnosed with breast cancer in the US last year) and second leading cause of cancer-related deaths, statistics that strongly advocate for a better understanding of the mechanisms that drive mammary carcinogenesis (Siegel et al., 2012). Cancers including breast cancer are initiated as a result of changes that occur in the genome. Differential gene expression analysis is commonly used to reveal the deregulated molecular mechanisms of complex diseases including cancer. Gene expression profiling has classified breast cancer into intrinsic subtypes (Carey et al., 2006, Sørlie et al., 2001). Among these is the basal-like subtype, representing ER/PR-negative with low HER2 expressing tumors are characterized as the triple negative breast cancer (TNBC). Thus, TNBC is defined by histopathologies as a subtype that lacks the expression of estrogen receptor (ER), progesterone receptor (PR), and HER2 amplification/overexpression (Dey et al., 2013a, Rastelli et al., 2010, Foulkes et al., 2010). TNBC accounts for approximately 15% of breast cancer diagnoses, but approximately 25% of breast cancer-related deaths due to a more aggressive biology and lack of targeted therapies (Grigoriadis et al., 2012, Dey et al., 2013b). Gene expression data has documented the heterogeneous nature of TNBC (Mayer et al., 2014).

Despite significant success of targeted anticancer therapies in ER + or HER2 + subtypes of breast cancers, patients with loco-regionally advanced or metastatic triple negative carcinoma have very limited therapy options, especially as chemo-resistance develops to standard chemotherapy. A lack of standardized markers that differentiate “basal-like” and TN subtypes underlines the heterogeneous nature of these cancers. Despite the unclear delineation between TN and “basal-like” they compose some of the worst prognoses in breast cancer, are associated with an undifferentiated metaplastic histology with stem cell-like characteristics and have a high incidence of metastasis (Lien et al., 2007, Ben-Porath et al., 2008, Honeth et al., 2008). Besides array-based gene expression analysis, a number of studies have reported genomic alterations that occur in TNBC clinical specimens and cell lines including comparative genomic hybridization and deep genomic profiling using next generation sequencing (NGS) technologies (Shah et al., 2012, Ding et al., 2010, Weigman et al., 2012, Loo et al., 2011, Neve et al., 2006, Fridlyand et al., 2006, Chin et al., 2006). Whole-genome sequencing of a single metastatic TNBC (mTNBC) patient's germline, primary tumor, metastatic tumor, and xenograft has also been reported, which showed the complexity of the somatic events that arise within a given TNBC (Ding et al., 2010). More recently, genome-sequencing studies of large subsets of retrospectively collected TNBC have been reported, which implicate *TP53*, *PIK3CA*, *NRAS*, *EGFR*, *RB1*, and *PTEN* (Shah et al., 2012, Cancer, 2012).

**Fig. 1:**
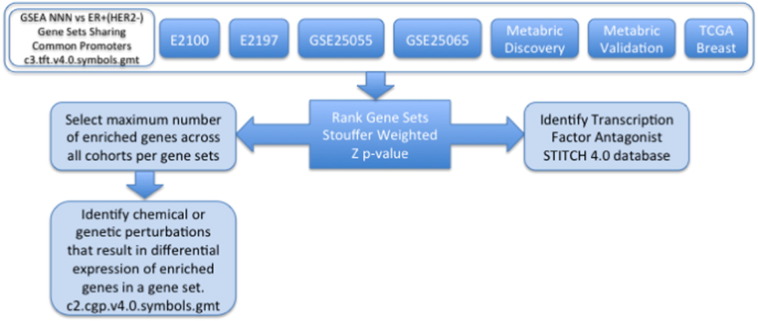
Each cohort consisting of TN and ER+(HER2 −) samples are run using GSEA to determine gene sets that are enriched and share a common promoter motif. The p-value from each enriched gene set is combined and ranked using Stouffer weighted Z to identify gene sets that have consistent enriched gene sets across all cohorts. The transcription factor for each ranked enriched gene set are searched in the STICH 4.0 database for chemical inhibitors or activators. Additionally, common set of genes in each gene set shown to be enriched across maximum number of cohorts are searched for known chemical and genomic perturbation gene set to identify possible inhibitors or activators.

To identify molecular mechanisms inherent to the TN subtype we have conducted gene set enrichment analysis (GSEA) (Subramanian et al., 2005), comparing TN vs. ER + HER2 −, in seven distinct cohorts, grouping gene sets by common promoter motifs to identify transcription factors and expression patterns of interest. The gene sets that are shown to be enriched in seven distinct cohorts with a Stouffer weighted Z (Whitlock, 2005, Zaykin, 2011) p-value < .01 are used to construct a promoter motif signature for genes determined to be enriched in the maximum number of cohorts. The transcription factor for each identified enriched promoter motif as well as any chemical or genetic perturbation that lowers the expression of the promoter motif gene signature represents potential therapeutic option(s) in TN breast cancer. The workflow is outlined in Fig. 1.

## Methods

#### Cohorts

Cohorts with representation of large N samples with immunohistochemistry (IHC) determined ER +/− and HER2 status and clinical outcome data were selected for analysis. All probe or gene expression levels were used as deposited using published normalization, and the following is a summary of each cohort. Each cohort is molecularly profiled on a wide range of platforms with different normalization methodology. GSEA is done independently for each cohort to determine statistically enriched gene sets mitigating the effects of different platforms and normalizations. The GEO deposited cohorts GSE25055 (n = 279 TN = 114/ER + 165) and GSE25065 (n = 187 TN = 64/123) were run on the U133A Affymetrix GeneChip with well-curated phenotype metadata and metastasis outcome (Hatzis et al., 2011). TCGA-BC RNA Seq V2 RSEM was downloaded from TCGA Data Portal on July 1, 2013 and represents (n = 286 TN = 58/ER + = 228) samples with IHC ER and HER2 metadata. Metabric Discovery (n = 413 TN = 69/ER + 344) and Metabric Validation (n = 236 TN = 52/ER + = 184) cohorts with frozen samples profiled on the Illumina V4 platform selecting for IHC determined ER subtype and HER2 = 1. Unpublished clinical trial cohorts, E2100 (n = 114 TN = 49/ER + = 65) (Miller et al., 2007) and E2197 (n = 573 TN = 191/ER + = 382) (Goldstein et al., 2008) representing FFPE samples profiled on Illumina Whole-Genome DASL with long term follow up, and IHC determined ER status and HER2 status were used in the analysis. E2197 cohort was cubic spline normalized using Illumina software. E2100 cohort was quantile normalized using Illumina software.

#### Probe and gene expression mapping

To provide for consistent gene names each platform assigned gene accession id or UniGene id was programmatically cross referenced to the HUGO recommended gene name. Probes with an identifier that had been withdrawn were removed from the data set. The probe with the maximum expression level for each gene in each sample was used to represent the transcription gene expression level.

#### Gene set enrichment analysis

IHC metadata for ER, PR and HER2 status was used to designate each sample TN or ER +. Samples that lacked corresponding IHC metadata were not included in the analysis. Each cohort has a range of metadata to classify a sample as TN or ER+(HER2 −). For NNN that indicates the first N = ER − status, second N = PR − status and third N = HER2 − status. An X indicates any value and in Metabric a 1 was used to indicate HER2 − status. GSEA was performed using Broad GSEA version 2.0.13 with default settings (Subramanian et al., 2005, Mootha et al., 2003) comparing TN vs ER + phenotype for enriched genes in the MSigDB gene set collection c3.tft.v4.0.symbols.gmt representing known gene sets sharing a common promoter motif.

#### Determining transcription factors and promoter motif signatures of interest

The GSEA determined p-value for each enriched gene set from each cohort was ranked using Stouffer weighted Z (Whitlock, 2005, Fisher, 1958) and ranked by p-value with a p-value < .01. For each statistically interesting gene set, it is expected that some number of genes will be consistently enriched in all cohorts. The enriched genes from each cohort were combined, and the genes found to be enriched in the maximum number of cohorts were used to make the promoter motif signature for each transcription factor gene set. The promoter motif signature can be used to determine chemical or genetic perturbations that down regulate the collection of enriched genes. For an enriched gene set that share a common promoter motif with a known transcription factor that transcription factor also becomes a potential therapeutic target.

#### Selection of possible therapeutic drugs

The MSigDB contains a unique gene set c2.cgp.v4.0.symbols.gmt that represents curated data of up or down regulated genes from 3402 chemical or genetic perturbation experiments. Each promoter motif gene signature was programmatically compared with each set of down or up regulated genes in c2.cgp.v4.0.symbols.gmt. Criteria stipulating 50% of the promoter motif enriched genes' signature was found in a chemically or genetically altered gene set then that c2.cgp.v4.0.symbols experiment indicates a mechanism to regulate the enriched genes sharing a common promoter motif. Experiments indicating the ability to down regulate the enriched genes are selected as being of interest.

For each enriched promoter motif with known transcription factor, the transcription factor was manually searched using the STITCH 4.0 database (Kuhn et al., 2014), which contains interactions between 390,000 small molecules and 3.6 million proteins from 1133 organisms. Drug interactions with the transcription factor that indicate inhibition or activation of the transcription factor are selected as being of interest.

## Results

The list of enriched gene sets sharing common promoter motifs in Table 1 is ranked by Stouffer weighted Z p-value, which combines probabilities from independent tests with cohorts of different sizes. A high degree of overlap occurs when genes share multiple common but distinct promoter motifs for a set of transcription factors and can be seen in Table 2. Genes that share different transcription factor promoter motifs are an example of the redundancy that allows for multiple mechanisms to drive the expression of that gene.

With the ranked list of enriched gene sets any combinations of genes could be enriched with minimal overlap between cohorts. To identify genes or promoter motif signatures each set of GSEA defined enriched genes per gene set are combined for all cohorts. Enriched genes found in the highest number of cohorts per gene set are combined and shown in Table 2. Genes found to be enriched in all seven cohorts are strong evidence that the genes sharing common promoter motifs and the corresponding transcription factor are possible therapeutic targets.

**Fig. 2:**
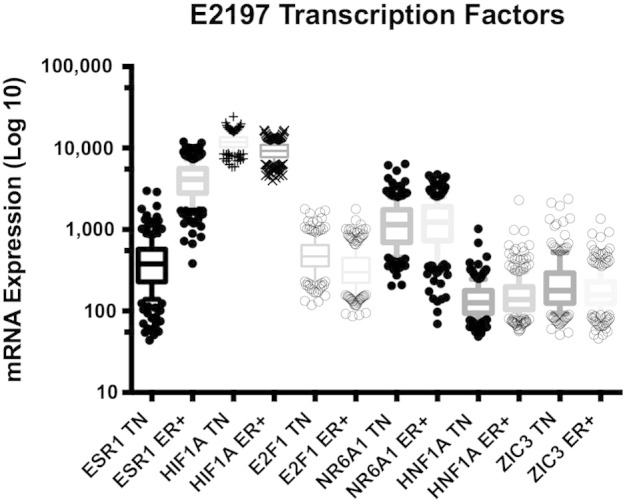
E2197 expression levels of ESR1 and NR6A1/GCNF, HNF1A, ZIC3 grouped by IHC determined TN or ER +. Expression levels of ESR1 establish an expected expression range. Box and Whisker plot with expression values outside of the 10–90 percentile indicated as black dots. ESR1 and HIF1A are shown for reference. Other genes are not expected to be expressed in normal tissue as indicated in Table 5. GCNF/NR6A1 has high expression in both TN and ER + samples where expression is only expected in germ cells of gonads.

Data mining of differential expression of mRNA in cancer cohorts and drug interaction databases can be tested in appropriate cell lines and mouse models for possible therapeutic benefit. This approach can identify drugs that are shown in published experiments and data sets to have properties as possible therapeutics in TN breast cancer. Additionally, examination of antagonists of transcription factors identified as potential drivers of enriched gene sets can increase the number of possible therapeutic targets. To identify possible therapeutic agents that can down regulate the expression of the promoter motif signatures found in Table 2, the signatures are compared to the c2.cgp.v4.0.symbols.gmt gene sets which contain a curated list of up or down regulated genes from 3402 published array studies and is shown in Table 3. This approach has the advantage of determining drugs that act through direct or indirect mechanisms that can down regulate the expression of genes enriched in TN. This is particularly important for promoter motifs with unknown transcription factors or transcription factors with no known antagonists. Additionally, transcription factors that are not expected to be expressed in a normal tissue are indicated in Table 5 and their expression patterns are shown in Fig. 2.

As a result of the data analysis, the RAS inhibitor salirasib is identified as a possible therapy as it down regulates mRNA expression for enriched genes sharing V$E2F_01, V$E2F_Q3 and KTGGYRSGAA_UNKNOWN promoter motifs (Blum et al., 2007). Listed in Table 3, the genes that have been shown to be down regulated by salirasib have a high concordance with genes shown to be enriched in TN cohorts sharing V$E2F_01, V$E2F_Q3 and KTGGYRSGAA_UNKNOWN promoter motifs suggesting a regulatory effect that may have a treatment benefit for the patient. By using genes enriched in TN cohorts that have been shown to be down regulated by salirasib may be an effective biomarker strategy for selecting patients to enroll in a salirasib clinical trial. The RAS inhibitor salirasib has been shown to impact transcriptional response across five different cancer cell lines effecting E2F-regulated and NF-Y regulated genes and the transcription factor FOS which control cell proliferation, blocks apoptosis, and induction of activating transcription factor BACH2 regulated genes which participate in translation and stress response (Blum et al., 2007).

Additionally, The EGFR inhibitor CL-387,785 down regulates enriched genes in the V$E2F_Q3 promoter motif signature indicating an additional therapeutic target. In non-small cell lung cancer, EGFR inhibitor- resistant gefitinib-treated and the (sensitive) CL-387,785-treated H1975 cells were compared to identify transcriptional changes with EGFR-activating mutations (Kobayashi et al., 2006). Aminopeptidase inhibitor Tosedostat was shown to down regulate enriched genes containing V$E2F_01 promoter motifs including MYC the transcription factor targeting V$MYC_Q2, V$MYCMAX_01 and V$MYCMAX_02 promoter motifs. Tosedostat down regulated five of nine enriched genes in the V$E2F_01 promoter motif signature in HL-60 cells (acute promyelocytic leukemia) (Krige et al., 2008) where down regulation of MYC could impact a large number of TN enriched genes. The SB216763 inhibitor of GSK3Beta treatment of RS4;11 human leukemia cell line, down regulated 474 genes at least 1.5 fold that were significantly enriched for genes related to cell cycle and MYC-regulated genes (Wang et al., 2010). Knockout experiments of TLX and BMP2 indicate a down regulation of V$E2F_01, KTGGYRSGAA_UNKNOWN and V$E2F_Q3 enriched genes implicating them as possible therapeutic targets (Lee et al., 2007, Zhang et al., 2008). E2F1 has minimal overall expression but does have higher expression in TN vs. ER + with a comparative p-value < 0.0001 (Mann–Whitney test).

In Table 4, each transcription factor was used to identify possible activating and inactivating drugs from the STITCH 4.0 database (Kuhn et al., 2014). Surprisingly, the identified enriched promoter gene sets had a single known transcription factor versus a family of transcription factors that target the same promoter. This is encouraging given that targeting single transcription factors reduces the off target effects of treatment. To identify transcription factors that have minimal expression in normal tissue the transcription factors were submitted to the gene expression barcode 2.0-web service, which determines if a gene is expressed in many tissues using Affymetrix HGU133plus2 array data (McCall et al., 2011). Identification of transcription factors that are not expected to be expressed in breast tissue or have highly specific tissue expression further implicates its value as a therapeutic target where the normal tissue expression profiles are shown in Table 5. Genes with expected expression in breast tissue were also found.

The enriched genes that share HIF1A (Hypoxia-inducible factor alpha subunit) with promoter motif V$HIF1_Q5 and V$HIF1_Q3 as the highest ranked transcription factor resulting in higher expression of genes in TN. HIF1A is expressed in many tissues and known to have mRNA and protein down regulated by mitoxantrone a TOP2 targeting drug (Toh and Li, 2011) and rapamycin a mTOR regulator and immunosuppressant (Wang et al., 2009). HIF1A has higher expression in TN vs ER + with a comparative p-value < 0.0001 (Mann–Whitney test). HNF1A targeting V$TCF1P_Q6 promoters with a Stouffer weighted Z p-value of .004 and ZIC3 targeting V$ZIC3_01 with Stouffer weighted Z p-value of .002 had a minimum number of enriched genes in common across all cohorts indicating lack of support for strongly enriched genes. ZIC3 is found to be expressed in cerebellum tissue with no known drug antagonist where a drug that blocks the actions of ZIC3 but does not cross the blood brain barrier should have minimal off-target effects. In Fig. 2, ZIC3 expression in TN is slightly higher than ER + with a comparative p-value = 0.015 (Mann–Whitney test) but overall expression is minimal compared to the other transcription factors. HNF1A also has overall low expression where TN samples do appear to have outliers and a comparative p-value = .018 (Mann–Whitney test).

Understanding expected normal tissue mRNA expression profiles can also be used to find novel targets without clearly defined functions or ligands. As an example, NR6A1 an orphan nuclear receptor with no known ligand where normal expression is found in germ cells of gonads and expressed in both TN and ER + samples from E2197 (Fig. 2) with no statistical difference. If the 10 TN enriched genes [ABTB2 AMD1 BCL11A BCL11B FGF9 ITGB8 MALL NFIB NFIL3 RASAL1] sharing the V$GCNF_01 promoter motif are important to disease progression then NR6A1 would be an ideal therapeutic target. Hirose et al. (Hirose et al., 1995) and Chen et al. (Chen et al., 1994) showed that NR6A1 mRNAs were predominant in mouse testis and were just detectable in the brain, liver, and lung. The effect of androgen manipulation to lower mRNA expression of NR6A1 was studied by Hu et al. (Hu et al., 2003) using Northern blot analysis. It has been suggested that gene expression in the testis and epididymis are regulated by a male steroid hormone, androgen (Cornwall and Hann, 1995). The expression levels of NR6A1 transcripts (2.3 and 7.4 kb) in epididymis increased gradually and reached a plateau 5–7 days after castration (Hu et al., 2003). A randomized clinical trial compared tamoxifen vs. tamoxifen and flouxymesterone (anabolic steroid with androgenic properties) in postmenopausal women with metastatic breast cancer where median time to progression was 199 days vs. 350 days respectively approaching statistical significance with a one sided p-value = .07 (Ingle et al., 1988). NR6A1 over-expression could be a potential biomarker for effective treatment with androgen therapy.

## Discussion

The genetic alterations in tumor cells are dynamic in driving cellular programming and resulting in mRNA expression profiles that are important in tumor fitness. New tools and technologies for genomic or systemic level analysis, as well as conventional biochemistry (including proteomic analysis) and cell biology approaches (phenotypic studies) are revealing how signaling pathways contribute to the development of cancer, as well as tumor evolution, dormancy and therapy-resistance. Here, we show by using GSEA comparing distinct TN vs ER + HER2 − cohorts a collection of genes that are enriched in TN that share common promoter motifs associated with distinct transcription factors.

Using GSEA it is possible to determine sets of genes with common attributes or features that are enriched in a specific cancer phenotype. With the growing collection of large N cancer cohorts, this type of comparative analysis can identify potentially important genes that drive phenotype. Ideally, GSEA could be used to compare a cancer phenotype cohort with the expression profile of the normal tissue type affected by the cancer. We lack established large N normal tissue cohorts that can be used in GSEA. Using enriched gene sets when comparing common cancer phenotypes can identify common attributes that can give insight into the mechanisms that drive cancer and serve as possible therapeutic targets. The enriched gene sets that are differentially expressed are most likely driven by a common set of transcription factors or transcription factor families, which can be used to determine the drivers of the cancer phenotype.

We have identified a collection of enriched genes that share a common promoter element that indicates a possible transcription factor that is driving the differential expression between two cancer phenotypes. We can validate or prioritize a particular transcription factor as a treatment target using expression profiles from normal tissue to understand the potential for off-target effects of therapy. Transcription factors that have minimal expression in normal tissues but are found to be differentially expressed or highly expressed in a cancer phenotype make an ideal candidate for further study. Additionally, with the large collection of publicly available experiments indicating differential expression of mRNA as a result of drug treatment we can identify potential therapies that can regulate the expression of enriched genes in a particular cancer phenotype. Exploratory analysis of mRNA cancer cohorts can identify data patterns that based on biological context can then be used to mine for possible therapies that can attenuate the expression pattern associated with a cancer phenotype. In Table 3, we identify from published experimental data possible therapies that can regulate enriched genes that share a common promoter motif where it may not be clear how those genes are regulated. In Table 4, a collection of possible drugs are identified that target transcription factors that have enriched genes in TN sharing a common promoter. In Table 5, we use the expression profile of the transcription factor in normal tissues to prioritize based on possible off-target effects.

The STITCH 4.0 database did not list a known E2F1 targeted therapy but we are able to discern from the chemical and genetic perturbation data sets that RAS inhibitor Salirasib, EGFR inhibitor CL-387785 and Aminopetidase inhibitor Tosedostat regulate genes with E2F1 promoters which are shown to be enriched in TN. NR6A1 is a member of the nuclear receptor superfamily of ligand-activated receptors that shares a common modular structure. These receptors play vital roles in development, cellular homeostasis, and cancer where over- or under-expression of some receptors has prognostic significance for patient survival. NR6A1 is normally expressed in germ cells of gonads making it an interesting therapeutic target in breast cancer where unexpectedly it has high expression in both TN and ER + E2197 samples.

The nuclear orphan receptors for which endogenous ligands have not been identified include nuclear receptor NR0B1 (adrenal hypoplasia congenita critical region on chromosome X gene), NR0B2 (small heterodimer partner), NR1D1/2 (Rev-ErbAα/β), NR2C1 (testicular receptor 2), NR2C2 (testicular receptor 4), NR2E1 (tailless), NR2E3 (photoreceptor-specific NR [PNR]), NR2F1 chicken ovalbumin upstream promoter transcription factor 1 (COUP-TFI), NR2F2 (COUP-TFII), NR2F6 (verbA-related protein), NR4A1 (Nur77), NR4A2 (Nurr1), NR4A3 (Nor1), and NR6A1 (GCNF). Results of receptor knockdown or overexpression in vivo and in cancer cell lines showed that orphan receptors exhibit tumor-specific pro-oncogenic or tumor suppressor-like activity (Safe et al., 2014). Nuclear receptors are expressed in multiple cancers and contribute to the cancer cell phenotype, and ligands (agonists or antagonists) for these receptors are important chemotherapeutic agents (Safe et al., 2011, Lee et al., 2011, Shi, 2007, Tobin and Freedman, 2006, Willson et al., 2000, Jordan, 2003a, Jordan, 2003b, Jordan, 2007, Jordan and O'Malley, 2007). The prognostic and functional roles of orphan receptors in cancer cells and their therapeutic implication especially in the solid tumors are not clear. A member of the family, Nur77 is highly expressed in both ER + and ER − breast tumors (Muscat et al., 2013), and the receptor appears to be highly expressed in more differentiated, low grade tumors (Alexopoulou et al., 2010). The examination of publicly available array data for lung tumors showed that high expression of Nur77 mRNA predicted increased patient survival (Safe et al., 2011), while high levels of Nur77 protein were found prognostic for decreased patient survival in another cohort study (Lee et al., 2012). NR6A1 has been found to be a transcriptional repressor, through specific binding to DR0 response elements, which is found in the Oct4 proximal promoter. Interestingly, NR6A1 can interact with DNA methylation proteins and is suggested to recruit DNA methylation complexes to repress and silence Oct4 expression (Wang and Cooney, 2013). Cancer stem cells play a critical role in the tumorigenesis of basal like breast tumors. Cancer stem cells are molecularly characterized based on the expression of various cell surface receptors including integrin a6 (CD49f), integrin b1 (CD29), hyaluronan receptor (CD44), and the stem cell self-renewal transcription factors NANOG, OCT4, and SOX2 (Zheng et al., 2013). Expression screening of cancer/testis genes in prostate cancer identified NR6A1 as a novel marker of disease progression and aggressiveness (Mathieu et al., 2013). In their study, Mathieu et al. reported the identification of 98 genes detected in castration-resistant prostate cancer CRPC, hormone-sensitive prostate cancer (HSPC) and testicular samples but not in the normal controls. Among them, cellular levels of NR6A1 were found to be higher in HSPC compared to normal prostate and further increased in metastatic lesions and CRPC. It is also reported that an increased NR6A1 immunoreactivity was significantly associated with a high Gleason score, advanced pT stage and cancer cell proliferation.

Our data in line with these reports strongly indicate that NR6A1 expression warrants further investigations in breast cancer subtypes, especially in women with TNBC. The functional and prognostic studies on orphan receptors in different tumor types are limited. The role of NR6A1 in tumors is poorly understood and requires additional research. Although a wealth of integrated molecular data exists in primary breast cancer, the data available are not exhaustive. Our data provide a framework for biological validation and experimentation that should guide preclinical studies. For those pathways with therapeutic targets currently under clinical investigation, these studies can be used as a catalog of molecular lesions potentially representing biomarker(s) of future treatment option or resistance to existing treatment.

## Figures and Tables

**Table 1:** Results of GSEA using gene set c3.tft.v4.0.symbols.gmt in seven different TN vs ER+(HER2 −) cohorts ranked by Stouffer weighted Z score. Each cohort has a range of metadata to classify a sample as TN or ER+(HER2 −). For NNN that indicates the first N = ER − status, second N = PR − status and third N = HER2 − status. An X indicates any value and in Metabric a 1 was used to indicate HER2 − status.

	E2100 NNN vs PXN	E2197 NNN vs PXN	GSE25055 NNN vs PXN	GSE25065 NNN vs PXN	Metabric discovery Nn1 vs PX1	Metabric validation Nn1 vs PX1	TCGA BRCA NNN vs PXN	Stouffer weighted Z
V$HIF1_Q5	0.428	0.002	0.002	0.103	0.006	0.041	0.003	4.98E − 09
V$HIF1_Q3	0.536	0.000	0.000	0.021	0.022	0.067	0.011	1.08E − 08
KTGGYRSGAA_UNKNOWN	0.810	0.000	0.024	0.031	0.034	0.008	0.030	1.86E − 07
V$E2F1_Q6	0.777	0.000	0.000	0.008	0.111	0.043	0.040	2.16E − 07
V$GNCF_01	0.000	0.000	0.016	0.124	0.020	0.047	0.338	4.34E − 07
V$HMGIY_Q6	0.006	0.010	0.028	0.051	0.057	0.028	0.029	9.29E − 07
MCAATNNNNNGCG_UNKNOWN	0.543	0.004	0.164	0.092	0.039	0.125	0.000	3.40E − 06
V$E2F_01	0.556	0.000	0.036	0.030	0.175	0.056	0.023	3.78E − 06
TTCNRGNNNNTTC_V$HSF_Q6	0.175	0.008	0.022	0.004	0.070	0.089	0.085	4.11E − 06
CTGRYYYNATT_UNKNOWN	0.008	0.056	0.055	0.061	0.024	0.066	0.032	1.02E − 05
V$MYCMAX_02	0.620	0.000	0.020	0.288	0.176	0.111	0.036	2.37E − 05
V$MYC_Q2	0.402	0.014	0.004	0.099	0.116	0.175	0.052	3.61E − 05
V$TAXCREB_02	0.570	0.032	0.037	0.160	0.047	0.032	0.051	5.53E − 05
CATTGTYY_V$SOX9_B1	0.281	0.030	0.080	0.017	0.045	0.084	0.118	6.33E − 05
V$E2F_Q3	0.606	0.002	0.029	0.059	0.249	0.167	0.071	7.11E − 05
V$AP1_Q4	0.428	0.008	0.185	0.471	0.052	0.018	0.063	9.65E − 05
MYAATNNNNNNNGGC_UNKNOWN	0.265	0.021	0.106	0.131	0.079	0.100	0.121	2.59E − 04
V$NFY_Q6	0.800	0.010	0.029	0.157	0.091	0.133	0.168	2.71E − 04
V$AP1_01	0.333	0.019	0.113	0.358	0.056	0.057	0.180	3.90E − 04
V$USF_C	0.756	0.008	0.152	0.252	0.275	0.095	0.014	5.34E − 04
CCAWYNNGAAR_UNKNOWN	0.713	0.028	0.048	0.189	0.454	0.119	0.003	6.90E − 04
V$NFKAPPAB_01	0.054	0.022	0.529	0.634	0.017	0.050	0.164	7.14E − 04
TGASTMAGC_V$NFE2_01	0.702	0.055	0.061	0.151	0.052	0.028	0.318	7.44E − 04
V$MYCMAX_01	0.823	0.015	0.187	0.413	0.246	0.069	0.008	8.60E − 04
TTGCWCAAY_V$CEBPB_02	0.420	0.010	0.097	0.181	0.120	0.293	0.180	8.64E − 04
V$TCF11MAFG_01	0.586	0.138	0.053	0.054	0.066	0.050	0.179	9.81E − 04
V$BACH2_01	0.230	0.035	0.173	0.243	0.097	0.164	0.120	0.001
V$ZIC3_01	0.050	0.079	0.080	0.325	0.096	0.237	0.157	0.002
RAAGNYNNCTTY_UNKNOWN	0.657	0.088	0.067	0.205	0.082	0.090	0.326	0.004
V$TCF1P_Q6	0.148	0.042	0.067	0.332	0.228	0.305	0.169	0.004
V$AP1FJ_Q2	0.202	0.065	0.256	0.385	0.158	0.148	0.132	0.006
V$AP1_Q2	0.253	0.146	0.116	0.376	0.158	0.141	0.088	0.006

**Table 2:** Promoter motif gene signatures that are enriched in the most cohorts indicating that genes are consistently enriched when comparing TN vs ER+(HER2 −) expression.

Gene Set	#Genes	#Cohorts	Genes Enriched in the Most Cohorts
CATTGTYY_V$SOX9_B1	21	7	[ANKRD11 BCL11A CDK8 CLIC4 EHBP1 FEN1 FGF9 ILF2 MID1 NASP NDRG2 NXN RBMS1 S100A1 SEPHS1 SFRP1 SLC6A9 TLE1 TRIM2 VLDLR YEATS2]
V$HIF1_Q3	18	7	[ATP1B3 BCL11A CA9 E2F3 EN1 ENO1 EPHB3 GPM6B IGF2BP3 KIAA0664 KLF11 LRP8 NFIL3 NPR3 ST6GAL1 VEGFA YBX1 YEATS2]
V$HMGIY_Q6	16	7	[ANKRD11 ATP1B3 BCL11A CXCL10 DLL3 DONSON EN1 FAM98A LMO4 MIA NDRG2 NMU PPP2R3A PTPN22 ST8SIA1 TFAP2C]
V$HIF1_Q5	13	7	[BCL11A CA9 E2F3 EN1 EPHB3 GPM6B IGF2BP3 KLF11 LRP8 NT5DC2 PIM1 VEGFA YBX1]
V$MYC_Q2	10	7	[CSDA FOSL1 FXYD6 IL15RA IVNS1ABP LRP8 PABPC4 REXO2 TAGLN2 YBX1]
TTCNRGNNNNTTC_V$HSF_Q6	8	7	[CRYAB E2F3 H2AFY2 LRP8 SLC15A2 TBPL1 TFCP2L1 VEGFA]
MYAATNNNNNNNGGC_UNKNOWN	7	7	[AMD1 BBOX1 CDC20 KRT9 PTK7 TLE1 TLE4]
TTGCWCAAY_V$CEBPB_02	7	7	[CEBPB CLDN10 KRT23 SOX10 SPIB SRPK1 SYNCRIP]
CTGRYYYNATT_UNKNOWN	6	7	[CDC42EP1 COL9A1 CRYAB DKK1 SMOX SOX10]
V$E2F1_Q6	36	6	[ARHGAP11A ATAD5 CDC25A CDK1 DMD DNMT1 E2F3 EFNA5 EHBP1 EPHB1 EZH2 FANCG FBXO5 GAPDH GINS3 GMNN GPRC5B H2AFZ KLF5 MCM3 MCM4 MCM6 MCM7 MYC NASP NCL NUP62 PIM1 POLD1 PRPS2 RANBP1 RBL1 RRM2 SYNCRIP TYRO3 WDR62]
V$E2F_Q3	28	6	[ARHGAP11A CDC25A CDC45 CDK1 DMD DNMT1 DPYSL2 E2F3 EPHB1 EZH2 FBXO5 GMNN KIF15 KLF5 MCM3 MCM4 MCM6 MCM7 NASP NPR3 NUP153 PIM1 PLK4 POLA2 RANBP1 TCP1 TIPIN USP1]
V$NFY_Q6	18	6	[ACTL6A BUB1 CDK1 CENPF CKS2 GART LRP8 MYO1E NEK2 PNOC PRR11 PTCH1 SFRP1 TLE4 TOP2A TTK UGP2 ZBTB5]
KTGGYRSGAA_UNKNOWN	12	6	[EZH2 MCM3 MCM6 MCM7 MYC PIM1 POLD1 RANBP1 RFC4 SYNCRIP TUBA4A YARS]
V$GNCF_01	10	6	[ABTB2 AMD1 BCL11A BCL11B FGF9 ITGB8 MALL NFIB NFIL3 RASAL1]
V$E2F_01	9	6	[AMD1 DNMT1 E2F3 MCM3 MCM4 MYC NASP RANBP1 SMC4]
MCAATNNNNNGCG_UNKNOWN	8	6	[AMD1 CDCA3 EPHB1 PIGA PPP2R5D SH3BGRL3 SUV39H1 ZFAND5]
V$TAXCREB_02	2	6	[BCL11A CRLF1]
V$NFKAPPAB_01	26	5	[CBX2 CCL5 CD40 CD70 CD83 CDK6 CXCL10 CXCL11 CXCL16 IL27RA ITGB4 LIX1L LTA MSN NFKB2 NFKBIB ORAI1 RBMS1 RELB SMOC1 SOX10 TNFRSF9 TRIM47 UBD WNT10A ZBTB5]
V$AP1_Q4	25	5	[ASS1 CA9 COL27A1 CORO1C EDN1 EPHA2 FGF11 GJB3 GPR3 IRAK1 KCNN4 KIAA0664 LAMB3 LAMC2 MAP2 MMP7 PIM2 REXO2 SLC4A11 STK40 TAGLN2 UBE2C UCHL3 USP13 XIRP1]
V$MYCMAX_01	22	5	[ATAD3A CSK EIF2C2 ESRRA GCSH GPM6B HMGA1 IVNS1ABP KCNN4 KIAA0664 LRP8 ODC1 PABPC1 PPRC1 PRDX4 RANBP1 REXO2 SLC6A15 SYNCRIP TIMM8A TUBA4A YEATS2]
V$AP1_01	20	5	[AQP5 CA9 CALB2 DMD ENO1 EPHA2 FGF9 FOSL1 GSTP1 IRAK1 LY6D MAP2 MMP7 PIM1 REXO2 S100A2 SERPINB5 TAGLN2 TINAGL1 TUBA4A]
V$TCF11MAFG_01	18	5	[CDC45 DYNC1I1 E2F3 ENO1 FGF9 FOSL1 IRX4 JOSD1 LMO4 NUDT11 PLS3 PPARGC1A PTCH1 REXO2 SEL1L3 SERPINB5 SOBP UCHL1]
TGASTMAGC_V$NFE2_01	12	5	[ANGPTL4 ASS1 CA9 CALB2 E2F3 FOSL1 GSTP1 MYO10 PLS3 S100A2 SERPINB5 TINAGL1]
CCAWYNNGAAR_UNKNOWN	10	5	[AKR1B1 ARL4C CDC45 CKS1B DMD FAM49A RBMS1 TBX19 UQCRH YBX1]
V$MYCMAX_02	18	4	[CALB2 DZIP1 EN1 FBL FRMD4A HDGF HIF1A IVNS1ABP KIAA0664 NOTCH1 PABPC4 PPRC1 PTCH1 PTK7 SOX10 SYNCRIP TNFRSF21 TYRO3]
V$USF_C	12	4	[ASS1 BATF3 EIF2C2 ETV4 FBL IVNS1ABP LRP8 PABPC4 SLC6A15 ST6GAL1 TNFRSF21 YBX1]

**Table 3:** Promoter motif signatures from Table 2 compared to chemical and genetic perturbation gene sets in c2.cgp.v4.0.symbols.gmt. The identified chemical or genetic perturbation is shown experimentally to down regulate the enriched genes in the Genes found column.

Chemical or genetic perturbations	CGP gene set	Genes found
Ras inhibitor Salirasib	BLUM_RESPONSE_TO_SALIRASIB_DN	**V$E2F_01** = [DNMT1 MCM3 NASP RANBP1 SMC4] V**$E2F_Q3** = [CDC25A CDC45 CDK1 DNMT1 EZH2 FBXO5 GMNN KIF15 MCM3 MCM6 NASP PLK4 POLA2 RANBP1 USP1] **KTGGYRSGAA_UNKNOWN** = [EZH2 MCM3 MCM6 POLD1 RANBP1 RFC4 SYNCRIP]
EGFR inhibitor CL-387785	KOBAYASHI_EGFR_SIGNALING_24HR_DN	**V$E2F_Q3** = [CDC25A CDC45 CDK1 EZH2 GMNN KIF15 MCM3 MCM4 MCM6 MCM7 PLK4 POLA2 RANBP1 TIPIN USP1]
Aminopeptidase inhibitor Tosedostat (CHR-2797)	KRIGE_RESPONSE_TO_TOSEDOSTAT_24HR_DN	**V$E2F_01** = [AMD1 MYC NASP RANBP1 SMC4]
SB216763 inhibitor of GSK3B	WANG_RESPONSE_TO_GSK3_INHIBITOR_SB216763_DN	**V$E2F_01** = [MCM3 MCM4 MYC NASP RANBP1] **KTGGYRSGAA_UNKNOWN** = [MCM3 MCM6 MCM7 MYC PIM1 RANBP1 TUBA4A]
Knockout TLX	ZHANG_TLX_TARGETS_60HR_DN	**V$E2F_01** = [AMD1 DNMT1 MCM3 MCM4 NASP SMC4] **KTGGYRSGAA_UNKNOWN** = [EZH2 MCM3 MCM6 MCM7 POLD1 RFC4 SYNCRIP]
Knockout BMP2	LEE_BMP2_TARGETS_DN	**V$E2F_Q3** = [CDC25A CDC45 DNMT1 E2F3 EZH2 GMNN KLF5 MCM3 MCM4 MCM6 MCM7 NASP NUP153 PLK4 RANBP1 TIPIN USP1] **V$E2F_01** = [AMD1 DNMT1 E2F3 MCM3 MCM4 NASP RANBP1]

**Table 4:** Transcription factors that target the identified enriched gene sets and drugs associated with inhibition and activation determined by STITCH 4.0.

Transcription factor	STITCH 4.0 drug inhibition	STITCH 4.0 activation	Gene set
HIF1A	Rapamycin	Oxygen, deferoxamine	[V$HIF1_Q3, V$HIF1_Q5]
E2F1		CD437, camptothecin	V$E2F1_Q6
NR6A1 GCNF	Androgen	DR-0, retinoic acid	V$GNCF_01
HMGA1	Mevalonate		V$HMGIY_Q6
MYC		Estrogen	[V$MYCMAX_02, V$MYCMAX_01]
JUN	TAM67	Adenosine triphosphate, rapamycin, troglitazone	[V$AP1_Q4,V$AP1_01,V$AP1FJ_Q2, V$AP1_Q2]
SOX9	Retinoic acid	Dexamethasone, androgen, DMOG	CATTGTYY_V$SOX9_B1
NFKB/NFKB1	15d-PGJ2, curcumin, aspirin	Adenosine triphosphate, aspirin	V$NFKAPPAB_01
NFE2		Vanadate	TGASTMAGC_V$NFE2_01
BACH2			V$BACH2_01
CEBPB	Retinoic acid	Retinoic acid	TTGCWCAAY_V$CEBPB_02
NFE2L1		Sodium arsenite	V$TCF11MAFG_01
TCF1/HNF1A			V$TCF1P_Q6
ZIC3			V$ZIC3_01
STAT1		Retinoic acid, V205	V$ISRE_01

**Table 5:** Determination of transcription factors are expressed in many tissues using Gene Expression Barcode 2.0 (McCall et al., 2011). Rows that are bold indicate transcription factors that are not normally expressed in breast tissue.

Gene	Genbank	Proportion	Entropy	High entropy	Tissues
HIF1A	NM_001530	0.916	0.289	Yes	Many
**E2F1**	**M96577**	**0**	**0**	**No**	**None**
**E2F1**	**NM_005225**	**0**	**0**	**No**	**None**
**GCNF**	**NM_001489**	**0**	**0.011**	**No**	**None**
**GCNF**	**X99975**	**0**	**0**	**No**	**None**
**GCNF**	**U80802**	**0**	**0.011**	**No**	**None**
**GCNF**	**AF004291**	**0.008**	**0.015**	**No**	**Testes**
**GCNF**	**N30069**	**0**	**0.004**	**No**	**None**
**GCNF**	**AA176289**	**0**	**0.004**	**No**	**None**
**GCNF**	**AW237089**	**0**	**0.012**	**No**	**None**
HMGA1	NM_002131	0.534	0.349	Yes	Many
HMGA1	AF176039	0	0.01	No	None
MYC	NM_002467	0.649	0.24	Yes	Many
MYC	BF514781	0	0.005	No	None
JUN	BG491844	0.947	0.087	No	Many
JUN	BC002646	0.16	0.251	Yes	Adipose_tissue breast breast_stroma breast_stroma:tumor bronchus cd31 + _cells cd49a + _cells cervix dorsal_root_ganglia endometrium lung myometrium ovary prostate_gland skeletal_muscle stomach_cardiac thyroid trachea trigeminal_ganglia urethra vagina
JUN	NM_002228	0.634	0.39	Yes	Many
JUN	BE327172	0.092	0.178	No	Breast_stroma breast_stroma:tumor bronchus cd49a + _cells fallopian_tube_epithelium head_and_neck_squamous_cell_carcinoma:tumor lung ovary parotid_gland thyroid trachea trigeminal_ganglia
SOX9	AI382146	0.695	0.222	Yes	Many
SOX9	NM_000346	0.679	0.293	Yes	Many
NFKB1	M55643	0.374	0.216	Yes	Many
NFE2	L13974	0.084	0.06	No	Blood bone_marrow cd14 + _cells lymphocytes macaca_mulatta_blood monocyte-derived_dendritic_cells monocyte neutrophils pbmc platelets stratagene_reference_rna
BACH2	NM_021813	0.076	0.121	No	Blood cd3 + _t_cells cd49a + _cells cd4 + _cells jurkat_cells lymphocytes macaca_mulatta_blood pbmc platelets tonsil
BACH2	AW450901	0	0.053	No	None
CEBPB	AL564683	0.977	0.162	No	Many
NFE2L1	AI361227	0.947	0.073	No	Many
NFE2L1	NM_003204	0.977	0.033	No	Many
NFE2L1	H93013	0.924	0.093	No	Many
**HNF1A**	**M57732**	**0**	**0**	**No**	**None**
**HNF1A**	**X71347**	**0**	**0.024**	**No**	**None**
ZIC3	NM_003413	0.008	0.006	No	Cerebellum
STAT1	NM_007315	0.916	0.333	Yes	Many
STAT1	BC002704	0.336	0.305	Yes	Many

## References

[bb0275] Alexopoulou A., Leao M., Caballero O., Da S., Reid L., Lakhani S. (2010). Dissecting the transcriptional networks underlying breast cancer: NR4A1 reduces the migration of normal and breast cancer cell lines. Breast Cancer Res..

[bb0055] Ben-Porath I., Thomson M.W., Carey V.J., Ge R., Bell G.W., Regev A. (2008). An embryonic stem cell-like gene expression signature in poorly differentiated aggressive human tumors. Nat. Genet..

[bb0150] Blum R., Elkon R., Yaari S., Zundelevich A., Jacob-Hirsch J., Rechavi G. (2007). Gene expression signature of human cancer cell lines treated with the ras inhibitor salirasib (S-farnesylthiosalicylic acid). Cancer Res..

[bb0100] Cancer G. (2012). Comprehensive molecular portraits of human breast tumours. Nature.

[bb0010] Carey L.A., Perou C.M., Livasy C.A., Dressler L.G., Cowan D., Conway K. (2006). Race, breast cancer subtypes, and survival in the Carolina Breast Cancer Study. JAMA.

[bb0200] Chen F., Cooney A., Wang Y., Law S., O'Malley B. (1994). Cloning of a novel orphan receptor (GCNF) expressed during germ cell development. Mol. Endocrinol..

[bb0095] Chin K., DeVries S., Fridlyand J., Spellman P., Roydasgupta R., Kuo W. (2006). Genomic and transcriptional aberrations linked to breast cancer pathophysiologies. Cancer Cell.

[bb0210] Cornwall G., Hann S. (1995). Specialized gene expression in the epididymis. J. Androl..

[bb0020] Dey N., Barwick B., Moreno C., Ordanic-Kodani M., Chen Z., Oprea-Ilies G. (2013). Wnt signaling in triple negative breast cancer is associated with metastasis. BMC Cancer.

[bb0040] Dey N., Young B., Abramovitz M., Bouzyk M., Barwick B., De P. (2013). Differential activation of Wnt-β-catenin pathway in triple negative breast cancer increases MMP7 in a PTEN dependent manner. PLoS One.

[bb0070] Ding L., Ellis M., Li S., Larson D., Chen K., Wallis J. (2010). Genome remodelling in a basal-like breast cancer metastasis and xenograft. Nature.

[bb0140] Fisher R.A. (1958). Statistical Methods for Research Workers.

[bb0030] Foulkes W., Smith I., Reis-Filho J. (2010). Triple-negative breast cancer. N. Engl. J. Med..

[bb0090] Fridlyand J., Snijders A., Ylstra B., Li H., Olshen A., Segraves R. (2006). Breast tumor copy number aberration phenotypes and genomic instability. BMC Cancer.

[bb0130] Goldstein L.J., O'Neill A., Sparano J.A., Perez E.A., Shulman L.N., Martino S. (2008). Concurrent doxorubicin plus docetaxel is not more effective than concurrent doxorubicin plus cyclophosphamide in operable breast cancer with 0 to 3 positive axillary nodes: North American breast cancer intergroup trial E 2197. J. Clin. Oncol..

[bb0035] Grigoriadis A., Mackay A., Noel E., Wu P., Natrajan R., Frankum J. (2012). Molecular characterisation of cell line models for triple-negative breast cancers. BMC Genomics.

[bb0120] Hatzis C., Pusztai L., Valero V., Booser D., Esserman L., Lluch A. (2011). A genomic predictor of response and survival following taxane–anthracycline chemotherapy for invasive breast cancer. JAMA.

[bb0195] Hirose T., O'Brien D., Jetten A. (1995). RTR: a new member of the nuclear receptor superfamily that is highly expressed in murine testis. Gene.

[bb0060] Honeth G., Bendahl P.O., Ringnér M., Saal L.H., Gruvberger-Saal S.K., Lövgren K. (2008). The CD44 +/CD24 − phenotype is enriched in basal-like breast tumors. Breast Cancer Res..

[bb0205] Hu Y., Zhou Z., Xu C., Shang Q., Zhang Y., Zhang Y. (2003). Androgen down-regulated and region-specific expression of germ cell nuclear factor in mouse epididymis. Endocrinology.

[bb0215] Ingle J., Twito D., Schaid D., Cullinan S., Krook J., Mailliard J. (1988). Randomized clinical trial of tamoxifen alone or combined with fluoxymesterone in postmenopausal women with metastatic breast cancer. J. Clin. Oncol..

[bb0250] Jordan V. (2003). Antiestrogens and selective estrogen receptor modulators as multifunctional medicines. 2. Clinical considerations and new agents. J. Med. Chem..

[bb0255] Jordan V. (2003). Antiestrogens and selective estrogen receptor modulators as multifunctional medicines. 1. Receptor interactions. J. Med. Chem..

[bb0265] Jordan V. (2007). SERMs: meeting the promise of multifunctional medicines. J. Natl. Cancer Inst..

[bb0260] Jordan V., O'Malley B. (2007). Selective estrogen-receptor modulators and antihormonal resistance in breast cancer. J. Clin. Oncol..

[bb0155] Kobayashi S., Shimamura T., Monti S., Steidl U., Hetherington C., Lowell A. (2006). Transcriptional profiling identifies cyclin D1 as a critical downstream effector of mutant epidermal growth factor receptor signaling. Cancer Res..

[bb0160] Krige D., Needham L., Bawden L., Flores N., Farmer H., Miles L. (2008). CHR-2797: an antiproliferative aminopeptidase inhibitor that leads to amino acid deprivation in human leukemic cells. Cancer Res..

[bb0145] Kuhn M., Szklarczyk D., Pletscher-Frankild S., Blicher T., von M., Jensen L. (2014). STITCH 4: integration of protein–chemical interactions with user data. Nucleic Acids Res..

[bb0170] Lee K., Jeong J., Wang J., Ma L., Martin J., Tsai S. (2007). Bmp2 is critical for the murine uterine decidual response. Mol. Cell. Biol..

[bb0230] Lee S., Li X., Khan S., Safe S. (2011). Targeting NR4A1 (TR3) in cancer cells and tumors. Expert Opin. Ther. Targets.

[bb0280] Lee S., Andey T., Jin U., Kim K., Singh M., Safe S. (2012). The nuclear receptor TR3 regulates mTORC1 signaling in lung cancer cells expressing wild-type p53. Oncogene.

[bb0050] Lien H.C., Hsiao Y.H., Lin Y.S., Yao Y.T., Juan H.F., Kuo W.H. (2007). Molecular signatures of metaplastic carcinoma of the breast by large-scale transcriptional profiling: identification of genes potentially related to epithelial-mesenchymal transition. Oncogene.

[bb0080] Loo L., Wang Y., Flynn E., Lund M., Bowles E., Buist D. (2011). Genome-wide copy number alterations in subtypes of invasive breast cancers in young white and African American women. Breast Cancer Res. Treat..

[bb0295] Mathieu R., Evrard B., Fromont G., Rioux-Leclercq N., Godet J., Cathelineau X. (2013). Expression screening of cancer/testis genes in prostate cancer identifies NR6A1 as a novel marker of disease progression and aggressiveness. Prostate.

[bb0045] Mayer I., Abramson V., Lehmann B., Pietenpol J. (2014). New strategies for triple-negative breast cancer—deciphering the heterogeneity. Clin. Cancer Res..

[bb0180] McCall M., Uppal K., Jaffee H., Zilliox M., Irizarry R. (2011). The Gene Expression Barcode: leveraging public data repositories to begin cataloging the human and murine transcriptomes. Nucleic Acids Res..

[bb0125] Miller K., Wang M., Gralow J., Dickler M., Cobleigh M., Perez E. (2007). Paclitaxel plus bevacizumab versus paclitaxel alone for metastatic breast cancer. N. Engl. J. Med..

[bb0135] Mootha V., Lindgren C., Eriksson K., Subramanian A., Sihag S., Lehar J. (2003). PGC-1alpha-responsive genes involved in oxidative phosphorylation are coordinately downregulated in human diabetes. Nat. Genet..

[bb0270] Muscat G., Eriksson N., Byth K., Loi S., Graham D., Jindal S. (2013). Research resource: nuclear receptors as transcriptome: discriminant and prognostic value in breast cancer. Mol. Endocrinol..

[bb0085] Neve R., Chin K., Fridlyand J., Yeh J., Baehner F., Fevr T. (2006). A collection of breast cancer cell lines for the study of functionally distinct cancer subtypes. Cancer Cell.

[bb0025] Rastelli F., Biancanelli S., Falzetta A., Martignetti A., Casi C., Bascioni R. (2010). Triple-negative breast cancer: current state of the art. Tumori.

[bb0225] Safe S., Kim K., Li X., Lee S.O. (2011). NR4A orphan receptors and cancer. Nucl. Recept. Signal..

[bb0220] Safe S., Jin U., Hedrick E., Reeder A., Lee S. (2014). Minireview: role of orphan nuclear receptors in cancer and potential as drug targets. Mol. Endocrinol..

[bb0065] Shah S., Roth A., Goya R., Oloumi A., Ha G., Zhao Y. (2012). The clonal and mutational evolution spectrum of primary triple-negative breast cancers. Nature.

[bb0235] Shi Y. (2007). Orphan nuclear receptors in drug discovery. Drug Discov. Today.

[bb0005] Siegel R., Naishadham D., Jemal A. (2012). Cancer statistics, 2012. CA Cancer J. Clin..

[bb0015] Sørlie T., Perou C., Tibshirani R., Aas T., Geisler S., Johnsen H. (2001). Gene expression patterns of breast carcinomas distinguish tumor subclasses with clinical implications. Proc. Natl. Acad. Sci. U. S. A..

[bb0105] Subramanian A., Tamayo P., Mootha V.K., Mukherjee S., Ebert B.L., Gillette M.A. (2005). Gene set enrichment analysis: a knowledge-based approach for interpreting genome-wide expression profiles. Proc. Natl. Acad. Sci. U. S. A..

[bb0240] Tobin J., Freedman L. (2006). Nuclear receptors as drug targets in metabolic diseases: new approaches to therapy. Trends Endocrinol. Metab..

[bb0185] Toh Y., Li T. (2011). Mitoxantrone inhibits HIF-1α expression in a topoisomerase II-independent pathway. Clin. Cancer Res..

[bb0285] Wang Q., Cooney A. (2013). Revisiting the role of GCNF in embryonic development. Semin. Cell Dev. Biol..

[bb0190] Wang W., Jia W., Xu G., Wang Z., Li J., Ma J. (2009). Antitumoral activity of rapamycin mediated through inhibition of HIF-1alpha and VEGF in hepatocellular carcinoma. Dig. Dis. Sci..

[bb0165] Wang Z., Iwasaki M., Ficara F., Lin C., Matheny C., Wong S.H.K. (2010). GSK-3 promotes conditional association of CREB and its co-activators with MEIS1 to facilitate HOX-mediated transcription and oncogenesis. Cancer Cell.

[bb0075] Weigman V., Chao H., Shabalin A., He X., Parker J., Nordgard S. (2012). Basal-like Breast cancer DNA copy number losses identify genes involved in genomic instability, response to therapy, and patient survival. Breast Cancer Res. Treat..

[bb0110] Whitlock M. (2005). Combining probability from independent tests: the weighted Z-method is superior to Fisher's approach. J. Evol. Biol..

[bb0245] Willson T., Brown P., Sternbach D., Henke B. (2000). The PPARs: from orphan receptors to drug discovery. J. Med. Chem..

[bb0115] Zaykin D. (2011). Optimally weighted Z-test is a powerful method for combining probabilities in meta-analysis. J. Evol. Biol..

[bb0175] Zhang C., Zou Y., He W., Gage F., Evans R. (2008). A role for adult TLX-positive neural stem cells in learning and behaviour. Nature.

[bb0290] Zheng Q., Banaszak L., Fracci S., Basali D., Dunlap S., Hursting S. (2013). Leptin receptor maintains cancer stem-like properties in triple negative breast cancer cells. Endocr. Relat. Cancer.

